# The Protective Effect of Camellia Bee Pollen on Alcoholic Fatty Liver in Zebrafish

**DOI:** 10.3390/nu18091454

**Published:** 2026-04-30

**Authors:** Xinyu Luo, Shujie Chen, Anjia Huang, Jingyi Zhang, Siyi Tian, Chenggang Cai, Ruiyu Zhu, Guiwei Rao

**Affiliations:** 1 College of Biological and Chemical Engineering, Zhejiang University of Science and Technology, Hangzhou 310023, China; 212303817025@zust.edu.cn (X.L.); 212403817018@zust.edu.cn (S.C.); hanj1546@163.com (A.H.); 222403857027@zust.edu.cn (J.Z.); 212203817005@zust.edu.cn (S.T.); zhuruiyu7@126.com (R.Z.); 2 College of Biological and Environmental Engineering, Zhejiang Shuren University, Hangzhou 310015, China

**Keywords:** camellia bee pollen, zebrafish, alcoholic fatty liver model, natural products

## Abstract

**Background/Objectives**: Camellia bee pollen refers to pollen pellets collected by bees from plant stamens and mixed with salivary secretions. Alcoholic fatty liver disease (AFLD), as the initial phase within the spectrum of alcohol-induced liver diseases, has resulted in a rising global incidence rate and treatment burden of such liver ailments. **Methods**: This study employs acute zebrafish juvenile and adult zebrafish chronic alcoholic liver models to explore the protective effects of camellia bee pollen as well as its ethanol and water extracts on zebrafish alcoholic fatty liver. **Results**: The research findings indicate that the intervention group treated with camellia bee pollen significantly mitigated the accumulation of lipid droplets in zebrafish larvae and notably improved the liver lobule structure of adult zebrafish, bringing it close to normal conditions. The camellia pollen intervention group could significantly decrease the levels of triglyceride (TG), total cholesterol (T-CHO), alanine aminotransferase (ALT), aspartate aminotransferase (AST), and malondialdehyde (MDA), while increasing the levels of glutathione (GSH) and total superoxide dismutase (T-SOD). **Conclusions**: This experiment indicates that the pollen of tea flowers has a significant protective effect against alcoholic liver damage.

## 1. Introduction

Alcoholic fatty liver disease (AFLD), serving as the initial phase of alcoholic liver disease (ALD), represents a metabolic hepatic disorder induced by prolonged and excessive alcohol intake. Alcoholism serves as a significant pathological factor contributing to liver parenchyma injury [1]. In recent years, concomitant with the rise in the frequency of alcohol consumption and the alteration of dietary structure among the Chinese population, the incidence of alcoholic liver disease (ALD) has been increasing annually [2]. The incidence of alcoholic liver disease is gradually increasing [3], thereby attracting extensive attention in the field of public health. Against this backdrop, screening for naturally occurring active ingredients with high safety to prevent and treat liver diseases has become a crucial research direction in contemporary drug development.

Camellia bee pollen refers to the granular material obtained through the processing of male reproductive cells from plant stamens, which are collected by bees and subsequently mixed with their glandular secretions [4]. The pollen of the tea tree bee contains over 250 bioactive components, such as polysaccharides, essential amino acids, vitamins, unsaturated fatty acids, phenolic compounds, flavonoid compounds, as well as various micronutrients, including Zn, Se, Fe, and Mg [5]. Known for its well-balanced nutritional profile, it offers notable health benefits. Furthermore, it demonstrates antioxidant properties, may help improve cardiovascular and cerebrovascular health, assist in lowering blood sugar and lipid levels, support prostate health, and contribute to regulating intestinal microbiota [6,7]. The existing literature primarily focuses on the effects of camellia bee pollen on non-alcoholic fatty liver disease or chemical liver injury. Nevertheless, the interactive mechanism among ethanol metabolism disorder, lipid deposition, and oxidative stress in AFLD has not been systematically explored.

Zebrafish (*Danio rerio*) demonstrates 87% genomic homology with humans, and its metabolic network features highly conserved key pathways among mammals. This evolutionary conservation renders it a crucial model for comprehending the molecular mechanisms underlying human diseases [8]. Research findings have indicated that the activation of PPAR α in the zebrafish liver can curtail the accumulation of triglycerides (TG) in the liver by suppressing the expression of genes associated with lipid production [9].

This study aims to evaluate the effects of camellia bee pollen (presented as ethanol extract, water extract and in its original form) on alcoholic fatty liver disease in acute alcoholic liver injury models and chronic alcoholic liver disease models. The acute alcoholic liver injury model in juvenile zebrafish and chronic alcoholic liver disease models in adult zebrafish were used to evaluate the protective effects of camellia bee pollen—in the form of ethanol extract, water extract, and raw material—on alcoholic fatty liver disease. The assessment was based on histological analysis of liver microstructure and relevant liver index data. The results indicate the hepatoprotective activity of Camellia bee pollen, providing an experimental basis for its high-value utilization in liver health applications.

## 2. Materials and Methods

### 2.1. Preparation of Camellia Bee Pollen Extract and Related Solutions

Camellia bee pollen was purchased from Youhui Bee Industry Co., Ltd. in Jiangshan, Zhejiang Province, China. A detailed analysis of the chemical composition will be presented in subsequent experiments on chemical separation and identification. After being ground, it was filtered through a 50-mesh sieve. The specific procedure for preparing the ethanol extract was as follows: The pollen was extracted with a 41% ethanol solution at a solid-to-liquid ratio of 1:30, followed by sonication at 40 °C for 121 min using an ultrasonic instrument (KQ-400KDB, Kunshan Ultrasonic Instrument Co., Ltd., Kunshan, China). The specific operation for obtaining the water extract was as follows: ultrasonic extraction was carried out at 43 °C for 92 min at a solid-to-liquid ratio of 1:30. Subsequently, the extract was filtered and concentrated into a paste via rotary evaporation using a rotary evaporator (RE-2000A, Shanghai Yarong Biochemical Instrument Factory, Shanghai, China). Finally, it was placed in a freeze-drying machine (SCIENTZ-10N/C, Ningbo Xinzhi Biotechnology Co., Ltd., Ningbo, China) for subsequent utilization. At present, this extraction process is confined to the laboratory scale, and there is a dearth of data regarding solvent recovery, energy consumption, and scalability feasibility. Consequently, it should not be considered to have direct industrial application potential.

In the preliminary single-factor experiments and response surface methodology experiments, the optimal extraction processes for the ethanol extract and water extract of camellia bee pollen were determined based on the total antioxidant capacity index. The optimal extraction conditions for the ethanol extract of camellia bee pollen were as follows: an ethanol concentration of 41%, an extraction time of 121 min, and an extraction temperature of 40 °C, resulting in a total antioxidant capacity of 1.28 mM, which was close to the theoretical value. The optimal extraction process for the water extract of camellia bee pollen was as follows: a solid–liquid ratio of 1:30 (g/mL), an extraction time of 92 min, and an extraction temperature of 43 °C, with a total antioxidant capacity of 0.78 mM, which was close to the theoretical value.

Camellia bee pollen ethanol extract: 500 mg of the freeze-dried camellia bee pollen ethanol extract was weighed and dissolved in 1 mL of absolute ethanol. Subsequently, 9 mL of distilled water was added to dilute it to a 50 mg/mL camellia bee pollen ethanol extract. After thorough homogenization, it was stored in a refrigerator at −20 °C for future utilization.

Camellia bee pollen water extract and aqueous solution: 500 mg of the freeze-dried camellia bee pollen water extract and 500 mg of the milled and sieved camellia bee pollen raw material (the raw material was originally purchased as bee pollen, without being subjected to extraction with alcohol or water. This raw material was used as a mixture between been pollen and water containing 50 mg of bee pollen per 1 milliliter of water) were, respectively, weighed. Then, 10 mL of distilled water was added to each to dissolve them into a 50 mg/mL camellia bee pollen water extract and aqueous solution. After being thoroughly mixed, they were stored in a refrigerator at −20 °C for subsequent application.

### 2.2. Zebrafish Treatment

A total of 2010 juvenile fish and 180 adult fish were used in this experiment, and the entire experiment lasted for 2 months. Preparation of anesthetic: Weigh 3.0 mg of tetracaine and dissolve it in 100 mL of distilled water. Store it at 4 °C for later use.

The zebrafish used in the juvenile fish experiment were wild-type zebrafish of the AB genotype (7 months old), obtained from the National Zebrafish Resource Center (Eugene, OR, USA). The zebrafish were reared in a temperature-controlled system at 28 °C, with a photoperiod of 10 h darkness and 14 h light. Fresh fish water was employed to preserve water quality. *Artemia nauplii* were fed to the zebrafish twice daily (at 9:00 and 18:00). The fish water was prepared by dissolving 8.88 g of calcium chloride, 4.93 g of magnesium sulfate heptahydrate, 2.59 g of sodium bicarbonate, and 0.23 g of potassium chloride in pure water, followed by dilution to 40 L, and the pH was stabilized at 7.8 ± 0.2. If required, the pH was adjusted using 0.1 M hydrochloric acid or sodium hydroxide.

Within the initial 12 h of embryo collection, adult zebrafish with a male-to-female ratio of either 1:1 or 1:2 were placed in spawning tanks and physically separated by partitions. At 8:00 the following day, the partition was removed to enable the zebrafish to engage in natural mating. At 9:00, the fertilized eggs were collected and transferred to the embryo culture medium. The temperature was kept constant at 28 °C, the culture medium was replaced every 24 h, and non-viable embryos were removed. The embryos were incubated for a duration of 72 h.

All animal rearing and experimental treatments were conducted in strict compliance with the regulations of the Institutional Animal Care and Use Committee (IACUC) of Zhejiang University of Science and Technology (approval No. 20240307) on 7 March 2024 (Hangzhou, China).

Using zebrafish at the 4 dpf (4 days post-fertilization) developmental stage, the acute ethanol exposure duration was set at 32 h [10]. A total of 4 dpf juvenile zebrafish were randomly transferred into a 6-well plate, with 20 juvenile fish placed in each well. The ethanol concentrations were set to 0.0, 0.5, 1.0, 1.5, 2.0, and 3.0%, with 3 wells per group. Following replacement with the embryo culture medium, the culture was continued in a 28 °C constant-temperature light incubator (LRH-70, Ningguo Shaying Scientific Instrument Co., Ltd., Ningguo, China) for 32 h, with the solution being replaced with a fresh one every 24 h. After observing and analyzing the mortality rate and deformity rate (including spinal curvature, spinal fracture, developmental delay, brain edema, and pericardial edema) of the juvenile fish, as well as the morphology of the liver, an appropriate ethanol concentration was selected to establish the model.

(1) Establishment of a juvenile zebrafish model of alcoholic fatty liver: zebrafish from the 4 dpf developmental stage were used, which were randomly allocated into two groups: the normal group and the model group. The normal group consisted of 150 fish, and the model group contained 1500 fish. The juvenile fish in the control group were immersed in the embryo culture medium, while those in the experimental group were immersed in the embryo culture medium supplemented with ethanol. Subsequently, they were incubated at 28 °C for 32 h [10]. The solution was refreshed every 24 h.

Specific grouping of zebrafish larvae: 150 normal zebrafish larvae were evenly and randomly distributed into 6-well culture plates as the control group (Group A); 1500 zebrafish larvae that received the 2% ethanol induction model were also evenly and randomly transferred to 6-well culture plates. The specific classification is as follows: Group B: model group; Group C: positive control group (26.25 mg/L of silybin); Groups D, E, and F: low, medium, and high concentration groups of tea tree flower ethanol extract (0.05, 0.1, 0.15 mg/mL of tea tree flower ethanol extract); Groups G, H, and I: low, medium, and high concentration groups of tea tree flower water extract (0.1, 0.3, 0.5 mg/mL of tea tree flower water extract); Groups J, K, and L: low, medium, and high concentration groups of raw materials of honey tea flower (0.05, 0.1, 0.3 mg/mL of tea flower honey raw materials). A total of 12 groups were formed, with 3 wells in each group. The control group and the model group were supplemented exclusively with embryo culture medium, while the other experimental groups were provided with embryo culture medium blended with corresponding concentrations of drugs or extracts. The larvae were cultivated in a 28 °C constant-temperature light incubator for 48 h, and the culture solution was renewed every 24 h. Finally, the zebrafish larvae were completely immersed in ice water for euthanasia. The liver tissues of the larvae were collected, and liver pathological sections were prepared. Various liver indicators (TG, T-CHO, GSH, ALT, AST, T-SOD, MDA) were measured to check the pathological changes of the liver.

(2) Establishing an adult zebrafish model of ethanol-induced fatty liver: This experiment used 3-month-old AB-type wild-type zebrafish and randomly divided them into two groups: a normal group and a model group. The normal group consisted of 30 zebrafish, and the model group comprised 150 zebrafish. Zebrafish in the normal group were immersed in fish culture water, whereas those in the model group were immersed in fish culture water containing 1% ethanol [11]. The fish culture water was replaced daily, and the zebrafish were cultured for 14 days.

The specific grouping of adult zebrafish: 30 normal zebrafish were randomly assigned to the control group (Group A). 150 zebrafish that were treated with 1% ethanol were randomly divided into 5 groups: Group B: model group (1% ethanol); Group C: positive control group (1% ethanol + 26.25 mg/L silybin); Group D: ethanol extract of camellia bee pollen group (1% ethanol + 150 mg/L camellia bee pollen ethanol extract); Group E: water extract of camellia bee pollen group (1% ethanol + 300 mg/L camellia bee pollen water extract); Group F: raw camellia bee pollen group (1% ethanol + 300 mg/L camellia bee pollen raw material). The fish’s breeding water was changed daily. After 14 days, the zebrafish were completely immersed in ice water for euthanasia. After the adult fish died, the liver tissues of the zebrafish were collected, liver pathological sections were prepared, and various liver indicators (TG, T-CHO, GSH, ALT, AST, T-SOD, MDA) were measured to check the pathological changes of the liver.

In the ethanol exposure experiment, each empty well of the six-well plate is regarded as an experimental unit. The treatment medium and environmental conditions in each well are completely identical. Consequently, each treatment group is composed of three independent wells (*n* = 3), which are employed for inter-group statistical comparisons. In the zebrafish larva experiment, the Petri dish functions as an experimental unit. The treatment medium and environmental conditions received by the individuals within each Petri dish are precisely the same. Therefore, each treatment group comprises three independent Petri dishes, where *n* denotes the sample size at the group level (*n* = 3) and is utilized for inter-group statistical comparisons. In the biochemical index experiment of the larva, liver tissues are harvested from each larva in the Petri dish, and a mixed sample from one Petri dish is processed. As a result, each Petri dish yields an independent biological replicate sample (three mixed replicate samples per group). In the zebrafish adult fish experiment, individual fish are considered the experimental unit. The liver is collected from independent fish (three fish per group), and a mixed sample from each group’s replicate sample is used for biochemical determination, as previously described. When imaging individual larvae/fish (e.g., using ORO staining), multiple individual samples are collected within the Petri dish/group. However, the statistical comparisons adopt the aggregated values at the Petri dish level (larvae) or the fish level (adult fish) to prevent pseudo-replication.

Anatomical procedures: Initially, position the fish in an inverted orientation (with the abdomen facing upward) beneath the dissection microscope. Employ a fine needle to secure it by piercing through the region below the fish’s caudal fin and operculum. Utilize a spring clip to incise the abdominal skin and muscles starting from the anterior part of the pelvic fin toward the operculum. Remove one side of the operculum, pectoral fin, and skin muscles to expose the body cavity. Once the body cavity is opened, the internal organs are revealed. The liver is situated at the anterior end of the body cavity, superior to the intestine. Grasp the connective tissue between the liver and the intestine using blunt-tipped forceps, and gently tear and separate it. Employ microscissors to meticulously excise the liver tissue from the target area while simultaneously identifying and removing the gallbladder, spleen, and swim bladder.

### 2.3. The Component Content of Camellia Bee Pollen

#### 2.3.1. Total Phenol Content

Following the method outlined by Francois et al. [12] and making appropriate modifications, 0.5 mL of the extract solution and 9.5 mL of distilled water were placed in a 25 mL volumetric flask, followed by the addition of 1 mL of Folin–Ciocalteu reagent. The mixture was shaken, and then 5 mL of sodium carbonate (7%, m/V) was added. After mixing, it was placed at room temperature in the dark for 20 min. The solution was then made up to 25 mL with distilled water, and the absorbance at 750 nm was measured. Using different concentrations of gallic acid standard solutions as the control, a standard curve was prepared, following the same procedure. The obtained linear equation was y = 5.7908x + 0.0466, with R^2^ = 0.9995. The results were expressed as the mg of gallic acid per g of extract powder.

#### 2.3.2. Total Flavonoid Content Polyphenols

The method outlined by Hossain et al. [13], with appropriate modifications, was used. Standard curve preparation: accurately weigh 0.02 g of rutin, and make up to 100 mL with 70% ethanol for later use (0.2 mg/mL). Aqueous solutions of 0, 0.5, 1.0, 1.5, 2.0, 2.5, and 3.0 mL of rutin standard solutions were aspirated into 7 capped test tubes, and water was added to make up to 5 mL. A total of 0.3 mL of 5% NaNO_2_ solution was added, shaken, and placed for 6 min. Then, 10% Al(NO_3_)_3_ solution of 0.3 mL was added. After shaking and standing for 6 min, 4 mL of NaOH solution (1 mol/L) was added, followed by 0.4 mL of distilled water, and shaken before being placed for 15 min. The absorbance was measured at 510 nm. The regression linear equation obtained was y = 3.3241x + 0.0454, with R^2^ = 0.9985. A total of 2 mL of the extract solution was precisely aspirated and tested according to the above steps. The total flavonoid content was calculated based on the equation. Each sample was tested in triplicate. The results were expressed as the mg of rutin per g of extract powder.

### 2.4. Oil Red O (ORO) Staining

Based on Zhou [10] and other relevant literature [14,15,16,17], an Oil Red O staining experiment was conducted: Samples of zebrafish were taken from each group and rinsed three times with phosphate-buffered saline (PBS). Then, they were immersed in 4% paraformaldehyde for a 10 min fixation treatment. After that, the fixative was replaced, and the zebrafish were fixed in a 4 °C refrigerator overnight. After the overnight fixation, the fixative was rinsed with PBS, and these samples were preserved in 85% and 100% glycerol solutions and underwent dehydration treatment with a shaker (TS-1102, Shanghai Tiancheng Experimental Instrument Manufacturing Co., Ltd., Shanghai, China) at a rotational speed of 120 revolutions per minute for 15 min. Then, the samples were placed in a 0.3% Oil Red O staining solution and soaked at room temperature. They were stored in the dark and left to stand in the vibration apparatus for 1 h for staining treatment. After 1 h, the Oil Red O staining solution was dried, and 30 min gradient non-illuminated decolorization treatments were performed using 100% and 85% glycerol solutions, followed by rinsing with PBS three times. Finally, the liver morphology characteristics and lipid droplet deposition were observed under a microscope (ECLIPSE Si RS, Nanjing Nikon Jiangnan Optical Instrument Co., Ltd., Nanjing, China). A total of 10 samples were randomly selected for photography. The staining intensity and liver size were converted into grayscale values and area measurement values using Image J 1.54g software. These grayscale values and area measurement values were used to comprehensively reflect the degree of fatty lesion.

### 2.5. H & E Staining

Following the method described by Zhou [10,15], the livers of zebrafish larvae and adult zebrafish in each group were washed thrice with PBS, dried to eliminate moisture, immersed in a 4% paraformaldehyde fixative solution, fixed overnight, and subsequently rinsed with PBS. Thereafter, the samples underwent a series of procedures, including dehydration, transparency treatment, wax immersion, embedding, and sectioning into 4-μm tissue sections. The sections were then affixed to glass slides and dried at 65 °C. Subsequently, they were immersed in xylene I, xylene II, and xylene III for 3 min each for dewaxing, then immersed in anhydrous ethanol I, anhydrous ethanol II, 95%, 90%, and 80% ethanol solutions for 2 min each. After that, the sections were stained with hematoxylin for 10 min, and the excessive dye was washed away with clean water. Differentiation was carried out using 1% hydrochloric acid–alcohol for 10–15 s, followed by rinsing with running water. Then, the sections were counterstained with eosin dye for 3 min. Each sample was randomly observed in 3 fields of view, and each group had 3 biological replicates. After dehydration and sealing, these sections were observed and photographed under a microscope (ECLIPSE Si RS, Nanjing Nikon Jiangnan Optical Instrument Co., Ltd.).

### 2.6. Determination of Liver-Related Indicators

From each experimental group, 20 juvenile fish livers or 3 adult fish livers were selected, and three replicates were established. These samples were stored in cryovials, dehydrated, and flash-frozen in liquid nitrogen prior to being transferred to a −80 °C freezer for long-term storage. During utilization, the samples were thawed on ice, and physiological saline was added at a ratio of weight (mg):volume (µL) = 1:9. The mixture was ground in an ice bath and sonicated to prepare a 10% tissue homogenate. For measurement, the sample was diluted with the standard diluent at a specific ratio, and then the instructions of the reagent kit were followed. Bicinchoninic acid (BCA), triglycerides (TG), total cholesterol (T-CHO), reduced glutathione (GSH), aspartate aminotransferase (AST/GOT), alanine aminotransferase (ALT/GPT), total superoxide dismutase (T-SOD) and malondialdehyde (MDA); all the test kits were provided by the Nanjing Jiancheng Biotechnology Research Institute (Nanjing, China).

### 2.7. Data Analysis

All the results were analyzed via Graphpad Prism 10.3.1 and SPSS software (Version 16.0). Prior to data analysis, the Shapiro–Wilk test and Levene’s test were employed to validate the normality and homogeneity of variance of the data, respectively. The sample differences were derived through one-way analysis of variance. A *p*-value less than 0.05 signified a significant difference, a *p*-value less than 0.01 denoted an extremely significant difference, and a *p*-value less than 0.001 indicated an extremely high-level significant difference. Subsequently, Tukey’s HSD post hoc test was further utilized for pairwise comparisons among groups.

## 3. Results

### 3.1. Camellia Bee Pollen Extraction Rate and Component Content

During sample preparation, the alcohol extraction rate was 22.66%, and the water extraction rate was 33.39%.

**Figure 1:**
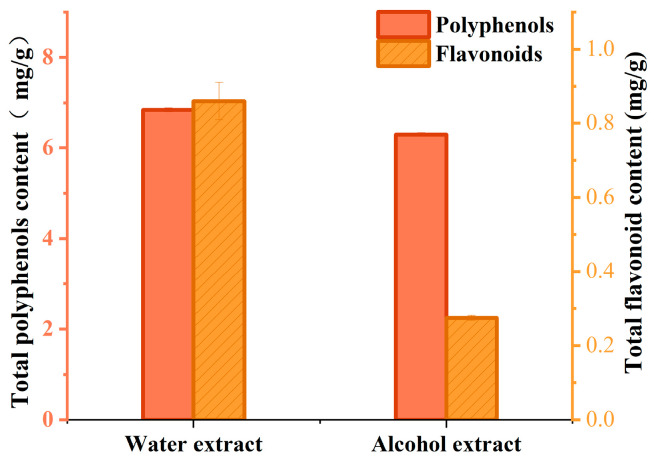
Content of polyphenols and flavonoids in water extract and alcohol extract.

As shown in Figure 1, the water extract has higher polyphenol and flavonoid contents than the alcohol extract. Based on this, it can be inferred that the water extract may have a better protective effect compared to the alcohol extract.

### 3.2. Determination of Ethanol Concentration in Zebrafish Fry Experiment

#### 3.2.1. Effects of Different Ethanol Concentrations on Mortality and Malformation Rates of Zebrafish Juveniles

Zebrafish larvae that had developed to 4 dpf were exposed to ethanol concentrations ranging from 0.5% to 3.0% for 32 h, while a control group was fed with embryo culture medium. In comparison with the control group, zebrafish fry exposed to an ethanol concentration of 3.0% exhibited a more rapid mortality rate. Specifically, within 32 h, the mortality rate reached 91.67%, and the malformation rate was 100%. Zebrafish fry exposed to 0.5% and 2.0% ethanol did not experience mortality within 32 h; however, the rate of developmental abnormalities increased significantly as the ethanol concentration rose (Table 1). Observation and data analyses were carried out using GraphPad Prism 10.3.1. The findings demonstrated that the median lethal concentration (LC_50_) of ethanol for zebrafish larvae was 2.89%, and a concentration of 2.0% could serve as a high-stress screening model for zebrafish larvae.

#### 3.2.2. Effects of Different Ethanol Concentrations on Hepatic Steatosis in Zebrafish Fry

**Figure 2:**
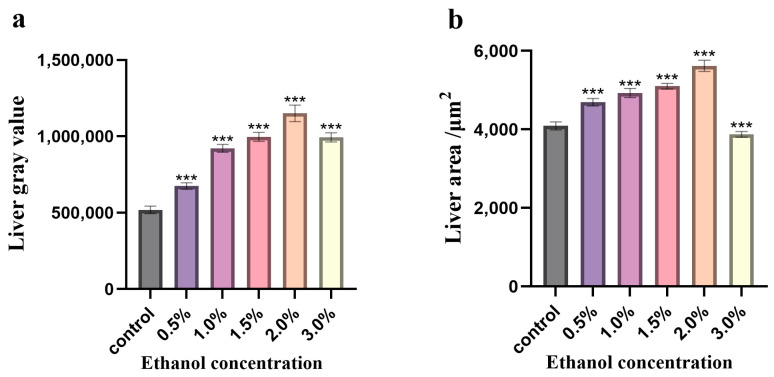
Effects of different ethanol concentrations on the gray value and area of zebrafish juvenile liver. (**a**): Liver gray value; (**b**): liver area. Note: compared with the control group, *** indicates *p* < 0.001, and the error bars represent the standard deviation (*n* = 3).

Oil red O staining demonstrated that, when compared to the blank control group, the liver area of zebrafish treated with 0.5–3.0% ethanol exhibited intensified Oil Red O staining. The grayscale value and area of the zebrafish liver augmented with the elevation of ethanol concentration ranging from 0.5% to 2.0% (Figure 2a,b), suggesting severe lipid droplet deposition and varying degrees of liver enlargement in the liver. This implies that ethanol treatment induces fatty lesions in the zebrafish liver. In contrast to the control group, a notable reduction in liver area and a substantial increase in grayscale value were detected in zebrafish treated with 3% ethanol. It is hypothesized that the liver may be in a pathological state of lipid metabolism disorder, accompanied by liver parenchymal damage or atrophy, resulting in lipid accumulation and a decrease in liver area. As depicted in Figure 2, the gray value and area value of the zebrafish liver treated with 2% ethanol are the highest, indicating the most severe degree of liver swelling and lipid accumulation. Based on the conclusion reached in the previous section, which indicates that a 2.0% ethanol concentration serves as the in vivo high-stress screening model for testing juvenile zebrafish, a 2% ethanol concentration is thus designated as the simulated concentration for the acute alcoholic fatty liver model of zebrafish larvae.

### 3.3. The Alleviating Effect of Camellia Bee Pollen on Acute Alcoholic Liver Injury in Zebrafish Fry

#### 3.3.1. Effects of Camellia Bee Pollen on the Liver Microstructure of Zebrafish Juvenile with Acute Alcoholic Liver Injury

**Figure 3:**
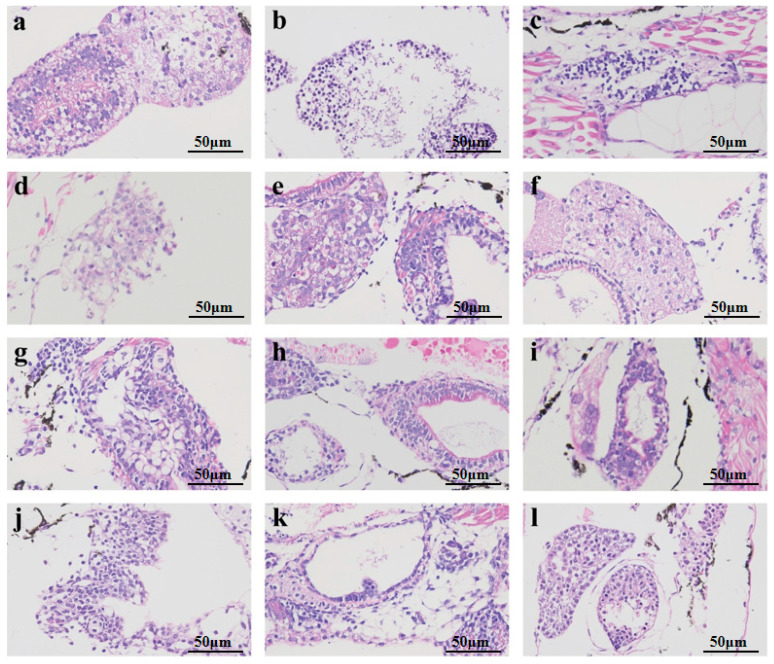
HE staining of liver tissue paraffin sections of zebrafish fry (×400). (**a**): Control group; (**b**): model group (2% ethanol); (**c**): positive control group (26.25 mg/L silybin); (**d**): low-concentration group of alcohol extract from camellia bee pollen (0.05 mg/mL); (**e**): medium-concentration group of alcohol extract from camellia bee pollen (0.1 mg/mL); (**f**): high-concentration group of alcohol extract from camellia bee pollen (0.15 mg/mL); (**g**): low-concentration group (0.1 mg/mL) of camellia bee pollen water extract; (**h**): medium-concentration group (0.3 mg/mL) of water extract of camellia bee pollen; (**i**): high-concentration group of water extract of camellia bee pollen (0.5 mg/mL); (**j**): low-concentration group of camellia bee pollen raw material (0.05 mg/mL); (**k**): medium-concentration group of camellia bee pollen raw material (0.1 mg/mL); (**l**): high-concentration group of camellia bee pollen raw material (0.3 mg/mL).

The liver tissue sections of the juvenile fish were treated using the hematoxylin–eosin (HE) staining method. Representative sections from each group were selected to visually assess the effects of camellia bee pollen on the pathological characteristics of the liver in juvenile fish with acute alcoholic liver injury (Figure 3). In the blank control group (Figure 3a), the hepatocytes exhibited uniform size, with a complete and well-organized structure. The cytoplasm was homogenous, the nucleus was spherical and centrally located, the nuclear membrane was distinct, the nucleo-cytoplasmic ratio was normal, and no nuclear condensation or fragmentation was observed. Occasionally, minute lipid droplets and vacuoles were dispersed within the cytoplasm, which was in line with physiological lipid metabolism.

Nevertheless, notable pathological alterations were detected in the liver of the model group (Figure 3b). The liver cells exhibited extensive swelling and disarray, presenting a loose and diffuse appearance. A multitude of large vesicular lipid droplets and vacuoles were present in the cytoplasm, which coalesced and compressed the nucleus toward the periphery. Some liver cells underwent apoptosis, leading to liver dysfunction, which was in accordance with the state of acute alcoholic liver injury. Following treatment with medication, in the high-concentration groups of silibinin (Figure 3c), camellia bee pollen alcohol extract (Figure 3f), water extract (Figure 3h,i), and raw material (Figure 3l), the quantity of lipid droplet vacuoles exhibited a significant reduction, the number of large vesicles declined, the distribution of lipid droplets became more homogenous, the nuclear translocation phenomenon was mitigated, the nuclear-to-cytoplasmic ratio approached the normal level, the arrangement of liver cells regained order, and pathological features such as inflammatory cell infiltration were notably diminished. Additionally, it was discovered that the high-concentration group (Figure 3f,i,l) exhibited a superior effect on liver cell repair compared to the low-concentration group (Figure 3d,g,j). This finding suggests that the protective effect of camellia bee pollen components on liver cells is dose-dependent. Furthermore, among these three components, the liver cell protection function of the water extract is stronger than that of the other components.

#### 3.3.2. Effects of Camellia Bee Pollen on Liver Indicators of Zebrafish Juvenile with Acute Alcoholic Liver Injury

**Figure 4:**
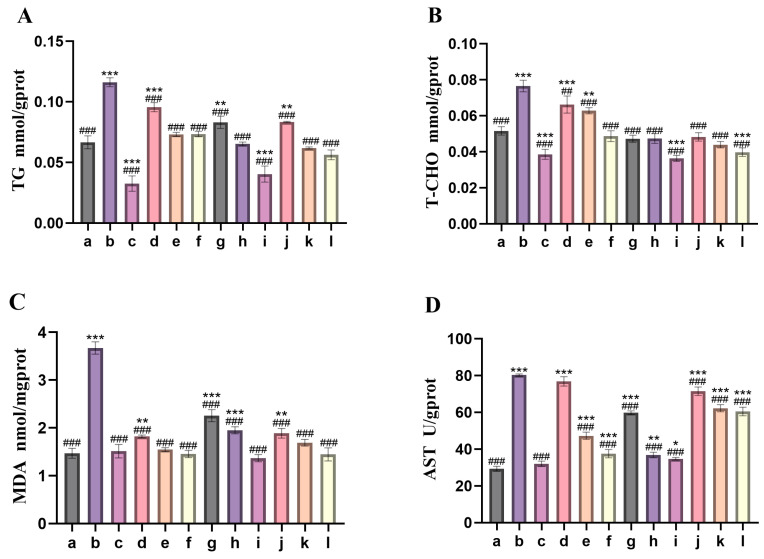

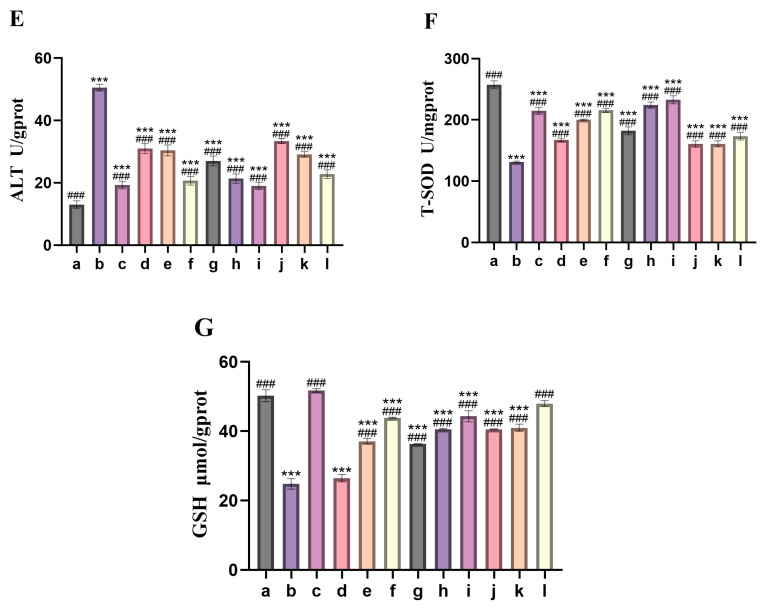
Various liver indicators of zebrafish fry. (**A**): Content of TG; (**B**): content of T-CHO; (**C**): content of MDA; (**D**): content of AST; (**E**): content of ALT; (**F**): content of T-SOD; (**G**): content of GSH. a: control group; b: model group (2% ethanol); c: positive control group (26.25 mg/L silybin); d: low-concentration group of alcohol extract from camellia bee pollen (0.05 mg/mL); e: medium-concentration group of alcohol extract from camellia bee pollen (0.1 mg/mL); f: high-concentration group of alcohol extract from camellia bee pollen (0.15 mg/mL); g: low-concentration group (0.1 mg/mL) of camellia bee pollen water extract; h: medium-concentration group (0.3 mg/mL) of water extract of camellia bee pollen; i: high-concentration group of water extract of camellia bee pollen (0.5 mg/mL); j: low-concentration group of camellia bee pollen raw material (0.05 mg/mL); k: medium-concentration group of camellia bee pollen raw material (0.1 mg/mL); l: high-concentration group of camellia bee pollen raw material (0.3 mg/mL). Compared with the control group, * *p* < 0.05, ** *p* < 0.01, *** *p* < 0.001; compared with the model group, ## *p* < 0.01, ### *p* < 0.001. The error bars represent the standard deviation (*n* = 3).

Figure 4 presents the measurement outcomes of the liver index for each group of zebrafish larvae. These data can be employed to assess the hepatoprotective effect of camellia bee pollen on acute alcoholic liver injury in zebrafish larvae. As depicted in Figure 4A,B, in comparison with the blank control group, the model group exhibited a notable elevation in the content of triglyceride (TG) and total cholesterol (T-CHO) (*p* < 0.001). The potential rationale could be that alcohol impeded the oxidation process of fatty acids, activated sterol regulatory element-binding protein-1c (SREBP-1c), facilitated lipid synthesis, resulted in fat accumulation, and initiated the development of fatty liver [18]. The activities of alanine aminotransferase (ALT) and aspartate aminotransferase (AST) significantly increased (*p* < 0.001) (Figure 4D,E). Alcohol-induced rupture of the liver cell membrane (ALT ↑) and mitochondrial damage (AST ↑) were detected. The ratio of AST/ALT is 1.59, which exceeds 1.5, suggesting the potential presence of mitochondrial dysfunction or the progression of fibrosis and liver cell damage [19]; the content of malondialdehyde (MDA) exhibited a significant increase (*p* < 0.001) (Figure 4C), whereas the content of reduced glutathione (GSH) and the activity of total superoxide dismutase (T-SOD) showed a significant decrease (*p* < 0.001) (Figure 4F,G). The potential reason is that alcohol metabolism generates reactive oxygen species (ROS), which results in lipid peroxidation (elevated malondialdehyde, MDA), exhaustion of antioxidant enzymes (superoxide dismutase, SOD) and glutathione (GSH), along with oxidative stress responses [20]. In comparison with the model group, the indicators of triglyceride (TG), total cholesterol (T-CHO), alanine aminotransferase (ALT), aspartate aminotransferase (AST), and MDA in the camellia bee pollen intervention group were significantly reduced, while the indicators of T-SOD and GSH were significantly elevated. This may inhibit lipid synthesis by activating the peroxisome proliferator-activated receptor alpha (PPARα)/adenosine monophosphate-activated protein kinase (AMPK) pathway [21] or by inhibiting sterol regulatory element-binding protein-1c (SREBP-1c) [22], thereby promoting lipid oxidation. It may also reduce enzyme leakage through anti-inflammatory methods (such as inhibiting tumor necrosis factor-α) or by protecting mitochondria (such as activating SIRT1/PGC-1α) [23]. It may also enhance the expression of antioxidant enzymes by activating the Nrf2 pathway or directly eliminating reactive oxygen species (ROS) [24]. Furthermore, it is clearly discernible that the alcohol extract, water extract, and raw camellia bee pollen materials exert a dose-dependent effect on the protection of the livers of zebrafish larvae with acute alcoholic liver injury. Specifically, a higher concentration corresponds to a more pronounced liver-protective effect. In comparison, the liver-protective effect of the alcohol extract is relatively feeble, whereas that of the water extract is more potent. The hepatoprotective effect of the high-concentration aqueous extract exhibits a greater resemblance to that of the positive control drug, and it demonstrates a more favorable inclination to ameliorate lipid metabolism disorders, oxidative stress, and liver damage. This conclusion is in accordance with the results of the microscopic structural analysis of the larvae’s livers in the previous section.

### 3.4. The Alleviating Effect of Camellia Bee Pollen on Alcoholic Liver Injury in Adult Zebrafish

#### 3.4.1. Effects of Camellia Bee Pollen on the Liver Microstructure of Adult Zebrafish with Alcohol Induced Liver Injury

**Figure 5:**
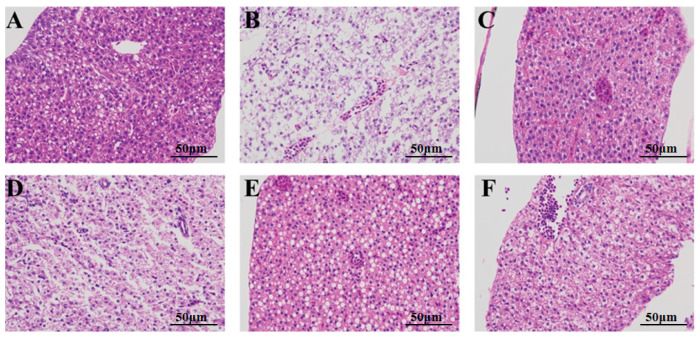
HE staining of paraffin sections of adult zebrafish liver tissue (×400). (**A**): control group; (**B**): model group (1% ethanol); (**C**): positive control group (26.25 mg/L silybin); (**D**): camellia bee pollen alcohol extract group (150 mg/L); (**E**): camellia bee pollen water extract group (300 mg/L); (**F**): camellia bee pollen raw material group (300 mg/L).

Representative liver tissue sections of adult zebrafish were selected for observation (Figure 5). It is observable that the liver cells in the blank control group (Figure 5A) exhibit a distinct and intact liver lobular structure. The hepatic cord cells are densely arranged in a radial pattern. The central veins and hepatic sinusoids display regular morphologies, without dilation or congestion. The liver cells are regular and orderly, with uniform cytoplasm. The nuclei are round and centrally located, featuring a clear nuclear membrane. The nuclear–cytoplasmic ratio is normal, and there is no nuclear condensation or fragmentation. Moreover, there is no infiltration of inflammatory cells, no hepatocyte necrosis or apoptotic bodies, and no ballooning degeneration or fatty degeneration. Occasionally, small lipid droplet vacuoles are scattered in the cytoplasm, which is consistent with physiological lipid metabolism. The model group, which was established by immersion in 1% ethanol (Figure 5B), exhibited a disordered hepatic lobule structure. The hepatic cords were loosely arranged, and the hepatocytes showed extensive swelling and disarray. In local areas, dissolution and necrosis of hepatocyte nuclei were observed. Moreover, a large number of large vesicular lipid droplets and vacuoles were present in the cytoplasm, pushing the cell nucleus to the periphery. The lipid droplets and vacuoles occupied over 50% of the cytoplasmic area of hepatocytes, suggesting severe steatosis, which was consistent with the state of alcoholic liver injury. The successful establishment of the model was verified.

Following drug treatment, the liver tissue of the positive control group (Figure 5C) exhibited a general restoration comparable to that of the blank control group. The hepatic lobular structure approximated that of the normal group, with hepatic cords arranged in an orderly manner. The morphology of central veins and hepatic sinusoids remained intact. There was a notable decrease in the quantity of lipid droplets and vacuoles, primarily transitioning from macro-vesicular to micro-vesicular forms, and the lipid droplets were more uniformly distributed. Nevertheless, inflammatory cell infiltration and a small quantity of necrotic lesions persisted. The pollen constituents of camellia bee also exert a protective influence on alcoholic liver injury. Among them, the protective effect of the alcohol extract (Figure 5D) is relatively weak. It can partially restore the structure of liver lobules, render the arrangement of hepatic cords denser compared to the model group, and decrease the quantity of lipid droplets and vacuoles in liver tissue. However, a small number of large vesicles still persist, accounting for approximately 20–30% of the cytoplasmic area, which is classified as moderate steatosis. Intervention with the alcohol extract can effectively mitigate lipid overload. The protective effect of the camellia bee pollen raw material (Figure 5F) is more potent than that of the alcohol extract. It restores the majority of the liver lobular structure, significantly reduces the number of lipid droplets and vacuoles, and the pattern changes from predominantly large-vesicular to micro-vesicular. The phenomenon of nuclear translocation is mitigated, and the nuclear-to-cytoplasmic ratio approaches the normal level. However, inflammatory cell infiltration and partial necrosis are still observable. In comparison with the control group, the water extract of camellia bee pollen exhibits a more potent protective effect. As depicted in Figure 5E, the liver lobule structure resembles that of the control group, with hepatic cords arranged in an orderly manner. The lipid droplets and vacuoles in the cytoplasm are predominantly microbubbles, with the nucleus located centrally and the nuclear membrane clearly defined. The nuclear–cytoplasmic ratio is normal, and the lipid droplets are uniformly distributed. Infrequent vacuole fusion, limited inflammatory infiltration, and an absence of necrotic lesions were observed. The experimental results indicated that the intervention effect of camellia bee pollen components was marginally weaker than that of the positive control, with the water extract exhibiting the most potent liver-protective effect. It is hypothesized that it can confer liver protection through the regulation of lipid metabolism (such as promotion of fatty acid beta-oxidation) or anti-inflammatory pathways [25].

#### 3.4.2. Effects of Camellia Bee Pollen on Liver Indicators of Adult Zebrafish with Alcohol Induced Liver Injury

**Figure 6:**
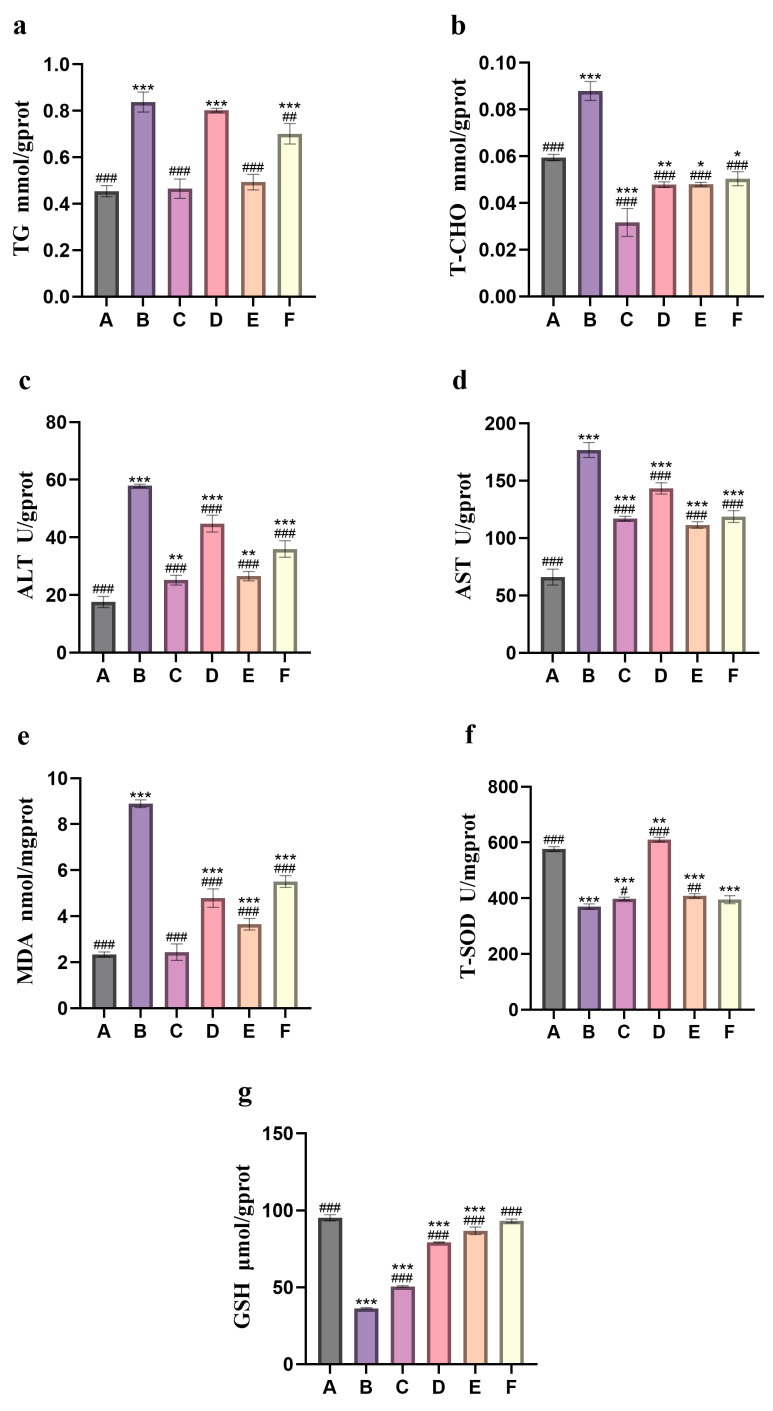
Liver indicators of adult zebrafish. (**a**): content of TG; (**b**): content of T-CHO; (**c**): content of ALT; (**d**): content of AST; (**e**): content of MDA; (**f**): content of T-SOD; (**g**): content of GSH. A: control group; B: model group (1% ethanol); C: positive control group (26.25 mg/L silybin); D: camellia bee pollen alcohol extract group (150 mg/L); E: camellia bee pollen water extract group (300 mg/L); F: camellia bee pollen raw material group (300 mg/L). Compared with the control group, * *p* < 0.05, ** *p* < 0.01, *** *p* < 0.001; compared with the model group, # *p* < 0.05, ## *p* < 0.01, ### *p* < 0.001. The error bars represent the standard deviation (*n* = 3).

Based on the liver index measurements of adult zebrafish in each group (Figure 6), when compared with the blank control group, the model group exhibited a notable elevation in lipid metabolism-related indicators, including triglycerides (TG) and total cholesterol (T-CHO) (Figure 6a,b), as well as liver function enzyme indicators such as alanine aminotransferase (ALT), aspartate aminotransferase (AST) (Figure 6c,d), and the level of the oxidative stress marker malondialdehyde (MDA) (*p* < 0.001) (Figure 6e). Conversely, the levels of total superoxide dismutase (T-SOD) and reduced glutathione (GSH), which are crucial components of the antioxidant defense system, decreased significantly (*p* < 0.001) (Figure 6f,g). Alcohol effectively induces lipid metabolism disorders, hepatocyte damage, and oxidative stress, leading to alcohol-induced liver injury. In comparison with the model group, the indicators of TG, T-CHO, ALT, AST, and MDA in the camellia bee pollen intervention group were significantly reduced, whereas the indicators of T-SOD and GSH were significantly elevated. Among these research findings, the aqueous extract exhibited a remarkable hepatoprotective effect, and its efficacy was even more comparable to that of the positive control drug. It is speculated that it may provide GSH synthesis precursors (such as N-acetylcysteine, NAC) [26] or upregulate the expression of synthetic enzymes (such as GCLC) [27]; directly enhance GSH biosynthesis; or inhibit ROS generation (such as inhibiting CYP2E1 activity) [28], reduce excessive consumption of GSH, enhance endogenous antioxidant defense, promote detoxification, inhibit inflammation and apoptosis, and have a more comprehensive protective effect on the liver.

## 4. Discussion

This research employed juvenile and adult zebrafish models to assess the protective effects of ethanol extract, water extract, and camellia bee pollen raw materials on alcoholic fatty liver damage. Juvenile zebrafish were subjected to an alcohol gradient treatment, and the half-lethal concentration (LC_50_) of juvenile zebrafish was ascertained based on the mortality rate and deformity rate, which was 2.89%. Subsequent to alcohol treatment, the livers of juvenile fish exhibited varying degrees of enlargement. However, in the fish treated with 3% ethanol, the liver area decreased while the gray value increased, suggesting a pathological state of lipid metabolism disorder. Consequently, the treatment method using 3% ethanol was excluded, and ultimately, a 2% ethanol concentration was chosen as the high-pressure screening model concentration for zebrafish larvae. An acute alcoholic fatty liver model was established through a 32 h soaking procedure. Please note: the larval arm is a severe screening model, whereas the adult model provides the stronger biological evidence.

In the juvenile zebrafish model, within the model group, the microscopic structure of the juvenile fish livers exhibited notable lipid degeneration and hepatocyte damage. Hepatocytes were swollen and disorganized, presenting a loose and diffuse appearance, and some hepatocytes underwent apoptosis. Conversely, in the intervention group treated with camellia bee pollen, the accumulation of lipid droplets was significantly diminished. The swelling of hepatocytes and nuclear displacement were alleviated. Moreover, the camellia bee pollen alcohol extract, water extract, and raw material groups demonstrated a dose-dependent liver cell protective effect. Among the three comparisons, the protective effect of the camellia bee pollen water extract was the most prominent. Analysis of different liver indicators revealed that in the model group of juvenile fish, the contents of triglyceride (TG) and total cholesterol (T-CHO) increased, the fatty acid oxidation capacity declined, and fat accumulation was observed [18]. The elevated activities of alanine aminotransferase (ALT) and aspartate aminotransferase (AST) suggest that there is damage to the liver cell membrane, mitochondrial dysfunction, and liver cells have been injured [19]. The content of malondialdehyde (MDA) increases, while the content of glutathione (GSH) and the activity of total superoxide dismutase (T-SOD) decrease. This indicates that there may be lipid peroxidation and oxidative stress responses [20]. After camellia bee pollen intervention in zebrafish, the indicators of TG, T-CHO, ALT, AST, and MDA significantly decreased, whereas the T-SOD and GSH indicators significantly increased. This indicates that the camellia bee pollen may be able to promote lipid oxidation, inhibit lipid synthesis, and enhance the expression of antioxidant enzymes. The alcohol extract, water extract, and raw tea bee pollen materials all exhibited notable hepatoprotective effects on juvenile zebrafish to different extents. With the increase in concentration, the protective effect became more prominent, showing a positive correlation. After comparison, it was found that the water extract showed a stronger protective effect, while the high-concentration water extract had the most significant liver-protective effect.

In the adult zebrafish model, this experiment employed a 1% ethanol concentration over a 14-day period to establish the model. Regarding the microscopic structure of the liver, the model group exhibited severe hepatic steatosis, hepatocyte swelling, lipid droplet accumulation, and liver dysfunction, accompanied by locally dissolved and necrotic hepatocyte nuclei. In contrast, the intervention group treated with camellia bee pollen significantly improved the structure of the liver lobules, reduced lipid droplet accumulation and inflammatory cell infiltration, and decreased the quantity of lipid droplet vacuoles, approaching the normal physiological state. A comparison of the protective effects among the alcohol extract, water extract, and raw material of camellia bee pollen revealed that the water extract had the strongest protective effect, yet it was still inferior to the positive control. Various liver indicators demonstrated that in the model group, triglyceride (TG), total cholesterol (T-CHO), and liver function enzymology indicators such as alanine aminotransferase (ALT), aspartate aminotransferase (AST), and malondialdehyde (MDA) all showed an upward trend, while total superoxide dismutase (T-SOD) and glutathione (GSH) levels showed a downward trend. This indicates that alcohol induced lipid metabolism disorders and alcoholic liver damage. After the intervention with camellia bee pollen, the TG, T-CHO, ALT, AST, and MDA indicators in the model group significantly decreased, while the T-SOD and GSH indicators significantly increased. It can be concluded that the water extract of camellia bee pollen has a better liver-protective effect, can enhance the endogenous antioxidant defense system, promote detoxification, inhibit inflammation and apoptosis, and exert a more comprehensive protective effect on the liver.

## 5. Conclusions

This research verified that camellia bee pollen exerts a notable protective effect on alcoholic liver damage in both acute and chronic zebrafish alcoholic liver models. It is noteworthy that the liver protective effect of the water extract is more prominent in both juvenile and adult zebrafish alcoholic liver models. By correlating the existing research results (lipid-lowering, antioxidant, and anti-inflammatory) with the known AFLD pathways, it was found that these indicators may be related to lipid metabolism (PPAR-α, SREBP-1c pathways) and antioxidant stress (Nrf2 pathways). Therefore, the underlying mechanism may encompass the regulation of lipid metabolism, the enhancement of antioxidant defense, and anti-inflammatory effects. These findings furnish experimental evidence for the high-value utilization of camellia bee pollen. Future research can further aim to incorporate standardized histopathological scoring methods to conduct a more objective and quantitative evaluation of the degree of liver tissue damage and to thoroughly explore the specific mechanism of action of camellia bee pollen and its clinical application prospects.

The zebrafish model provides a valuable in vivo experimental platform for mechanism exploration and large-scale screening. Moreover, many pathways related to liver lipid metabolism, oxidative stress, and inflammation are shared among vertebrates. The current research results should be regarded as preclinical evidence supporting the hepatoprotective effect of camellia bee pollen and its extracts. Further experiments are needed in mammalian models to investigate the effective oral dosage, absorption situation, and standardized processing methods, and ultimately to verify clinical efficacy in human studies.

## Figures and Tables

**Table 1:** Effects of different ethanol concentrations on mortality and malformation rates of zebrafish fry.

Ethanol Concentration (%)	Mortality Ratea (%)	Malformation Ratea (%)
0	0.00 ± 0.00	0.00 ± 0.00
0.5	0.00 ± 0.00	0.00 ± 0.00
1.0	0.00 ± 0.00	20.00 ± 5.00 **
1.5	0.00 ± 0.00	48.33 ± 10.41 ***
2.0	0.00 ± 0.00	80.00 ± 5.00 ***
3.0	91.67 ± 2.89 ***	100.00 ± 0.00 ***

Note: a represents three independent experiments. Compared with the control group, ** *p* < 0.01, *** *p* < 0.001 (*n* = 3).

## Data Availability

Data were provided in the manuscript.
